# Supplementary material for: Biopolymer from Water Kefir as a Potential Clean-Label Ingredient for Health Applications: Evaluation of New Properties
Source: Molecules. 2022 Jun 17;27(12):3895. doi: 10.3390/molecules27123895 (PMC9231297; doi:10.3390/molecules27123895)
Supplement: Supplementary file 1 [file molecules-27-03895-s001.zip › molecules-1703874-supplementary.pdf]

Biopolymer from Water Kefir as a Potential Clean-Label Ingredient for Health Applications: Evaluation of New Properties

<u>Supplementary Materials</u>

2.2 Evaluation of the growth rate (GGR) of water kefir grains

$$GGR\ (\%) = \frac{Final\ grain\ mass - Initial\ grain\ mass}{Initial\ grain\ mass}\ x\ 100 \qquad \text{(Eq.S1)}$$

2.6 Functional properties

2.6.1 Emulsifying ability (EA) and emulsifying stability (ES)

$$EA\ (\%) = \frac{Initial\ emulsion\ volume}{Total\ volume}\ x\ 100 \qquad \text{(Eq.S2)}$$

$$ES\ (\%) = \frac{Final\ emulsion\ volume}{Initial\ emulsion\ volume}\ x\ 100 \qquad \text{(Eq.S3)}$$

2.6.2 Water holding capacity (WHC)

$$WHC\ (\%) = \frac{Sample\ mass\ after\ water\ absorption}{Dry\ sample\ mass}\ x\ 100 \qquad \text{(Eq.S4)}$$

2.6.3 Water solubility (Sol)

$$Sol\ (\%) = \frac{Supernatant\ mass\ after\ drying}{Dry\ sample\ mass}\ x\ 100 \qquad \text{(Eq.S5)}$$

2.6.4 Foaming ability (FA) and foam stability (FS)

$$FA\ (\%) = \frac{Initial\ foam\ volume}{Total\ suspension\ volume}\ x\ 100 \qquad \text{(Eq.S6)}$$

$$FS\ (\%) = \frac{Final\ foam\ volume}{Total\ suspension\ volume}\ x\ 100 \qquad \text{(Eq.S7)}$$

2.6.5 Swelling index

$$Int\ (g/g) = \frac{Mass\ of\ swollen\ sample - Mass\ of\ dried\ sample}{Mass\ of\ dried\ sample} \qquad \text{(Eq.S8)}$$

2.7 Photostability study

$$\%\ \text{photodegradation} = [(A_0 - A_1)/A_0)] \times 100 \qquad \text{(Eq.S9)}$$

Where: A_0 is the absorbance at the wavelength at the initial time, and A_1 is the absorbance at the wavelength at the irradiation time.

$$\eta_{sp} = \eta_{rel} - 1 \qquad \text{(Eq.S10)}$$

$$\eta = \frac{\sqrt{2}}{C}(\eta sp - ln\ \eta rel)^{1/2} \qquad \text{(Eq.S11)}$$

Where *sp* and *rel* correspond to the specific and relative viscosity, respectively, and *C* corresponds to the concentration in g. dL^{-1}.

Table S1. Functional properties of EPSwk.

**Functional properties**	**Results**
Sol (%)	53.7 ± 2.0
EA (%)	89.3 ± 4,0
ES (%)	83,6 ± 1.8
WHC (%)	704.7 ± 53.3
FA (%)	26.7 ± 2.9
FS (%)	18.3 ± 4.0
Int (g/g)	43.1 ± 16.0

Sol - water solubility; EA - emulsifying ability; ES - emulsifying stability; WHC - water holding capacity; FA - foaming ability; FS - form stability; Int. - swelling index.

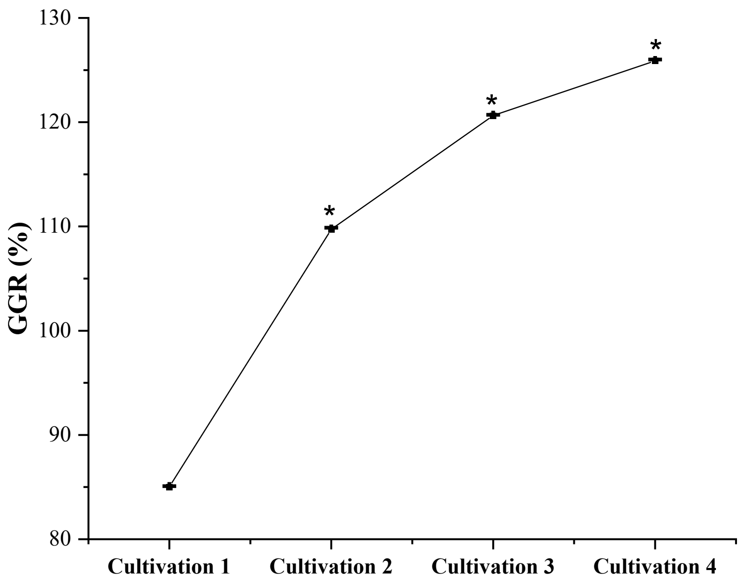

Figure S1. Monitoring the growth rate of water kefir grains in natura. * $p < 0.05$ compared to Cultivation 1 by the *Student's t-test*.

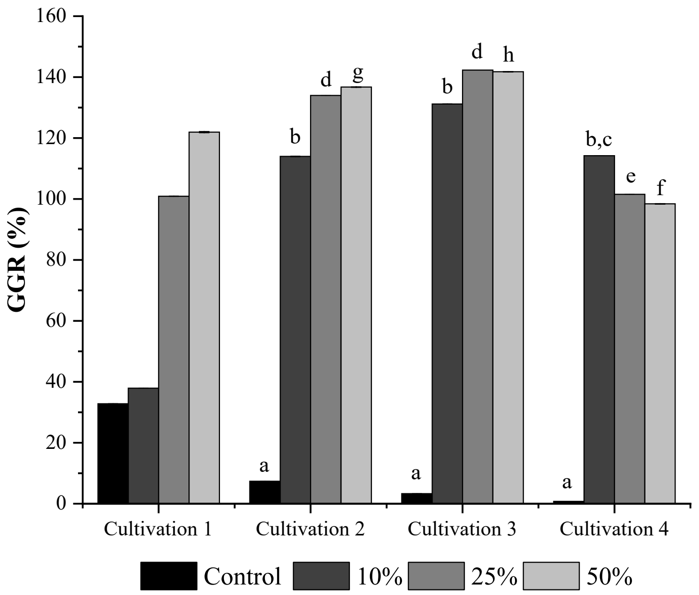

Figure S2. Monitoring the growth rate of water kefir grains after the freezing process with the addition of different concentrations of glycerol. a) $p < 0.05$ compared to control of the cultivation 1 by the *Student's t-test.* b) $p < 0.05$ compared to 10% glycerol of the cultivation 1 by the *Student's t-test.* c) $p < 0.05$ compared to 10% glycerol between cultivations 3 and 4 by the *Student's t-test.* d) $p < 0.05$ compared to 25% glycerol of the cultivation 1 by the *Student's t-test.* e) $p < 0.05$ compared to 25% glycerol among cultivations 2 and 3; 2 and 4; and 3 and 4 by the *Student's t-test.* f) $p < 0.05$ compared to 50% glycerol between cultivation 1 and 4 by the *Student's t-test.* g) $p < 0.05$ compared to 50% glycerol between cultivations 2 and 4 by the *Student's t-test.* h) $p < 0.05$ compared to 50% glycerol between cultivations 3 and 4 by the *Student's t-test.*

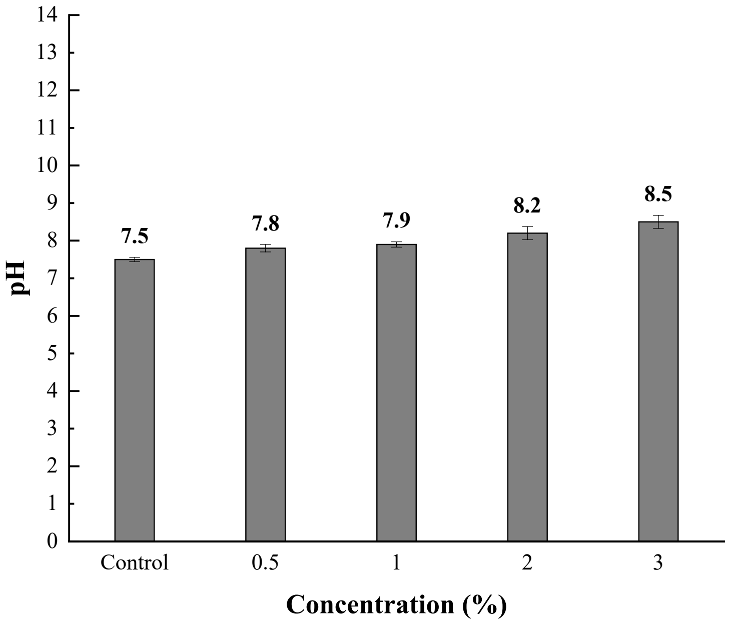

Figure S3. pH values of EPSwk in different concentrations.

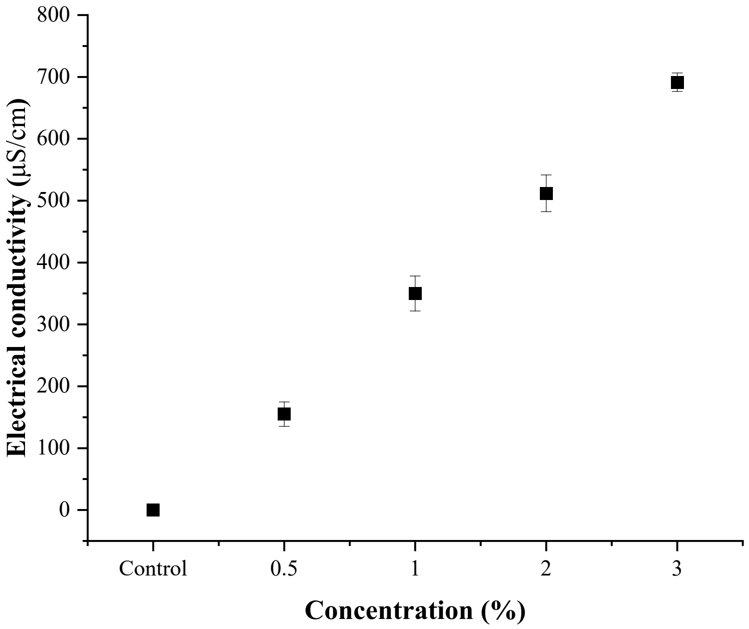

Figure S4. Electrical conductivity of EPSwk in different concentrations.

(a) **(b)**

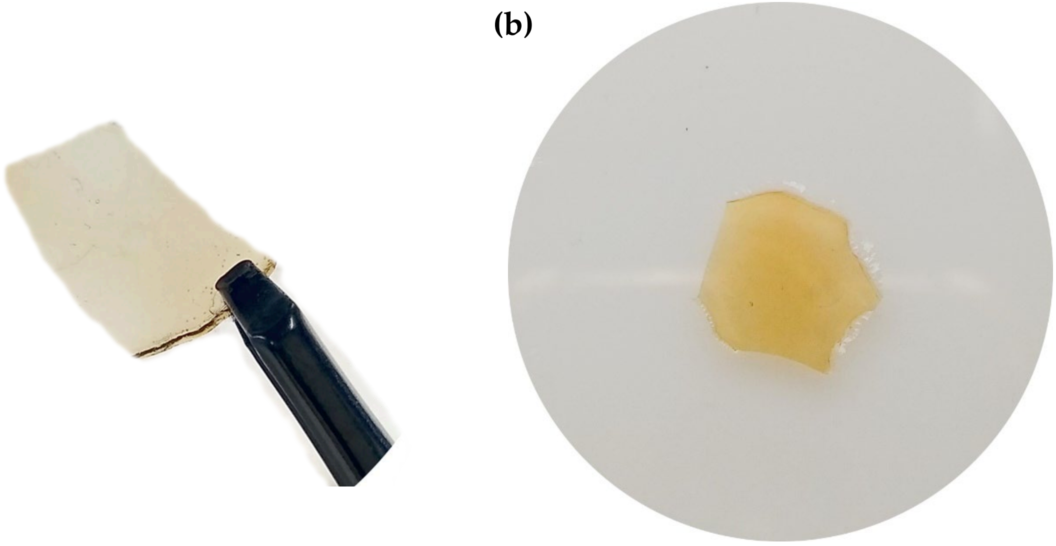

Figure S5. (a) Film of EPSwk without the addition of plasticizer. **(b)** Film of EPSwk with 10% of glycerol.

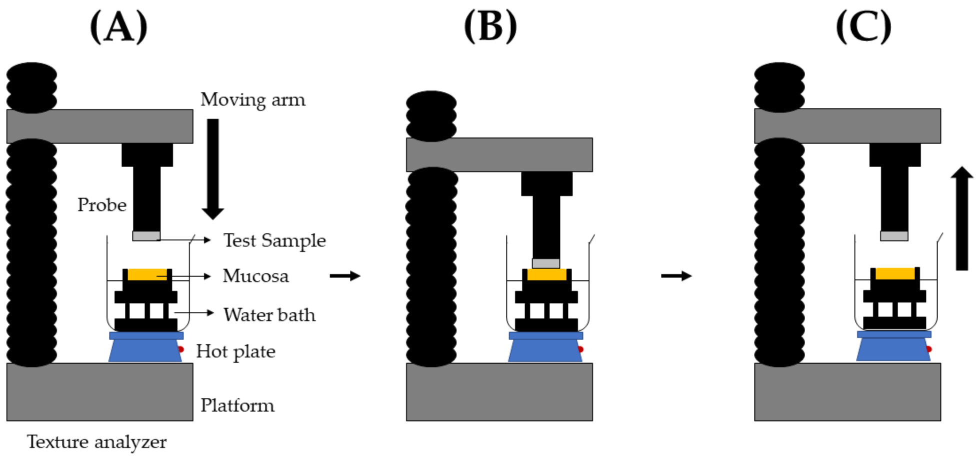

Figure S6. Schematic diagram of the mucoadhesiveness test by the texture analyzer. (A) The probe with the sample was moved downward; (B) The sample was attached to porcine buccal mucosa; (C). The probe moves up at a specified speed.

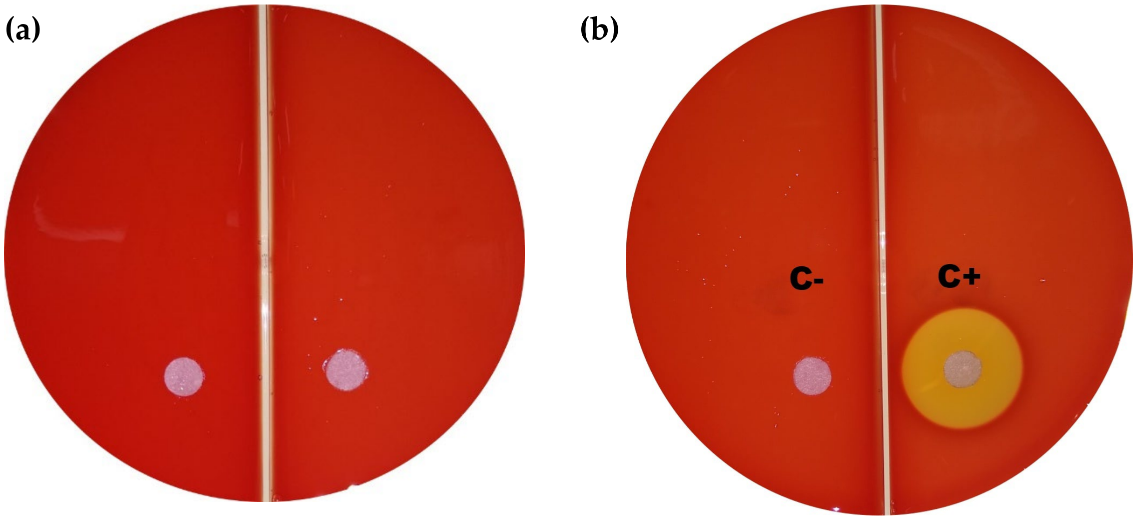

Figure S7. **(a)** Hemocompatibility analysis of EPSwk. **(b)** Negative and positive controls.
